#  Mitochondria and Familial Predisposition to Breast Cancer

**DOI:** 10.2174/1389202911314030005

**Published:** 2013-05

**Authors:** Stefania Weigl, Angelo Paradiso, Stefania Tommasi

**Affiliations:** National Cancer Research Centre, Istituto Tumori “Giovanni Paolo II”, Bari-Italy

**Keywords:** Mitochondrial DNA, Hereditary breast cancer, DNA variations, Polymorphism, Mitochondrial haplogroup.

## Abstract

Mitochondrial genome and functional alterations are related to various diseases including cancer. In all cases, the role of these organelles is associated with defects in oxidative energy metabolism and control of tumor-induced oxidative stress. The present study examines the involvement of mitochondrial DNA in cancer and in particular in breast cancer. Furthermore, since mitochondrial DNA is maternally inherited, hereditary breast cancer has been focused on.

## INTRODUCTION

Mitochondria are small cytoplasmic organelles which produce cellular energy in the form of ATP molecules by oxidative phosphorylation (OXPHOS) and contain several copies of mitochondrial DNA (mtDNA). The mitochondrial genome, a 16,569 bp circular double-stranded molecule, encodes for 2 ribosomal RNAs (rRNAs), 22 transfer RNAs (tRNAs) and 13 protein coding genes (PCGs) [1]. mtDNA is more strongly subjected to nucleotide modifications than the nuclear genome, showing a rate supposed to be from 10 to 20 times higher than nuclear DNA [2, 3]. It was recently suggested that the high mutation rate in mtDNA may result from the small effective population size which is associated with effectively haploid inheritance [4]. An important aspect of these mutations is mtDNA organization within the dynamic mitochondrial network in protein–DNA complexes known as “nucleoids”, which are involved in DNA replication, repair, gene expression, segregation, and inheritance [5].

Indeed, mitochondrial alterations, which range from severe to mild missense mutations, are rapidly fixed by several mechanisms such as genetic random drift and/or natural selection [6, 7]. Moreover, the mitochondrial genome is not protected by histones, and some molecular alterations, including thymidine dimers, cannot be repaired by mtDNA repair systems [8].

It has also been hypothesized that the high rate of reactive oxygen species (ROS) is a condition that probably encourages cancer development [9-11]. Severe mtDNA mutations could inhibit OXPHOS and promote tumor development, while milder mutations could permit a cancer to adapt to a new environment [12]. Moreover, mtDNA mutations seem to play other roles in carcinogenesis, creating a tissue susceptibility to cancer and/or a metastatic potentiality [13].

Several studies have demonstrated that many cancers harbour somatic mutations which are involved in mitochondrial dysfunction [14-16]. An alteration in mitochondrial function can change the developmental and replicative status of the nucleus, influencing the glucose metabolic pathway and deregulating the cell cycle of the surrounding stromal cells [17, 18]. Indeed, the uptake of glucose is highly increased in cancer cells, and the metabolic pathway results in a greater percentage of piruvate which is converted to lactic acid and excreted as a cathabolic product. This metabolic pattern is referred to as aerobic glycolysis, also commonly known as the “Warburg effect”. [18]. The activity of specific tumor suppressors (i.e., protein p53) decreases glycolysis and stimulates mitochondrial respiration through the activation of specific proteins required for the assembly of the citochrome c oxidase (*COX*) complex. Thus, the loss of p53 results in an increasing glycolysis and a decreasing mitochondrial respiration, contributing to the Warburg effect [19].

It has also been shown that cellular aging can act by accumulating mitochondrial DNA alterations, enhancing the production of ROS and representing a critical factor in the tumoral process [20]. The incidence of cancer significantly increases with human aging because of a progressive decline of mitochondrial function [21]. It has been reported that cell cycle arrest upon inhibition of mitochondrial function in human cells is under control of the mitochondria damage checkpoint, also known as the *mitocheckpoint* control [10]. The *mitocheckpoint* permits cells to arrest the cell cycle in order to normalize mitochondrial function. Severe damage of mitochondria may allow cells to undergo senescence, which represents the last checkpoint before the cell decision to start apoptosis or, alternatively, tumorigenesis [10]. 

Mitochondria are known for their role in mediating the intrinsic apoptotic pathway, also known as “mitochondrial apoptosis”. It has been demonstrated that apoptosis occurs less frequently in cells harboring mitochondrial alterations regarding those genes involved in the apoptotic process, such as the ATP synthase subunit 6 gene (*ATP6*), supporting the idea that pathogenic mtDNA mutations seem to promote tumors by preventing apoptosis [22]. Additionally, apoptosis can also be mediated by an extrinsic apoptosis pathway which is started by an activation of the cell-surface tumor necrosis factor family named “death” receptors. Both intrinsic and extrinsic apoptoses are cross-linked and converge upon the activation of caspases which carry out the final apoptotic steps [23]. Consequently, a discrete number of therapeutic strategies have recently been based on targeting tumoral mitochondria in order to induce the apoptotic way [24-27].

Since mitochondria play an essential role in the control of tumor-induced oxidative stress, the integrity of the mitochondrial genome and its functionality is strongly involved to contrast the oncogenic process [28]. Among the extensive typologies of human cancers, hereditary breast cancer is the most diffused tumour amongst the female population worldwide and it has captured the greatest attention among the scientific community. In the last years many authors have investigated both the contribution of nuclear alterations as increasing factors of hereditary predisposition to breast cancer [29-31] and the several mitochondrial alterations retrieved in breast cancer samples as possible susceptibility markers of the carcinogenic process [32-37]. This review focuses on how genetic background could play a critical role in modifying an individual risk to breast cancer, and why modifications in mtDNA could be highly involved in breast cancer progression.

## MITOCHONDRIAL DNA ALTERATIONS IN HUMAN CANCERS

The importance of mtDNA in cancer has been confirmed by the exchange of cancer cell mtDNA with pathogenic or normal mtDNA, resulting in alterations of cancer cell phenotypes [9, 11, 22]. Some recent insights have suggested that mtDNA mutations could be technical artifacts, due to nucleus-embedded mitochondrial sequences (NUMTs) ranging in size from ~50 bp to >15 kb [38, 39]. NUMTs, relocalized from the mitochondria to the nucleus during evolution, start to accumulate polymorphic mutations due to genetic drift [40, 41]. NUMTs are able to enter the nucleus especially under conditions of stress, as in case of tumorigenity. Since they have high similarity to authentic mtDNA, depending on the choice of primers NUMTs could be selectively amplified by PCR along with authentic mtDNA, allowing the misattribution of the mutations to those present in authentic tumoral mtDNA [42, 43]. These hypotheses are speculative and should not ignore the possibility that authentic mtDNA is mutated in tumors. Moreover, even if mutated DNA is an artefact derived from amplified NUMTs, it represents a useful marker of cancer and thus may have a clinical use [44-46].

In the past years, the whole human mtDNA or specific regions have been sequenced in many tumoral samples, revealing a very high frequency of mitochondrial alterations in various tissues and bodily fluids [32, 47, 48]. Mitochondrial DNA aberrations, including point mutations, instability of mono- or dinucleotide repeats, insertions, and deletions or quantitative alterations, have been found in solid tumors, such as breast, colon, head and neck cancer, stomach, liver, kidney, bladder, prostate, skin, lung cancer and several blood cancers [12, 35, 49].

In recent years, many studies have reported several mutations detected both in PCGs and in the main noncoding region, the displacement loop (D-loop). As the D-loop is responsible for replication control and transcription of the mitochondrial genome, mutations in this region modify mitochondrial genomic expression, deregulating mitochondrial metabolism and OXPHOS [50, 51]. In literature, some studies suggest how somatic mutations in the D-loop are involved in human carcinogenesis [52, 53]. For example, several studies reported that T146C, C150T and T152C polymorphisms occurred in tumor cell lines [54, 55]. Particularly, D-loop C150T was significantly indicated for an increased risk of cervical cancer and human papilloma virus (HPV) infection in Chinese women [56]. Moreover, the mitochondrial mutation T6777C, detected within cytochrome *c *oxidase subunit 1 (*CO1*), has been significantly linked to epithelial ovarian cancer [55]. It was also observed that elevated expressions of both *CO1 *and NADH dehydrogenase subunit 4 (*ND4*) were associated with gastric tumorigenesis and tumor dedifferentiation *ex vivo* [58]. An interesting study highlighted that a specific mtDNA mutation (A15296G) retrieved in cytochrome b (*CYTB*) was clonally detected in clinical samples of a leukemia patient, suggesting that this marker could play a role in cancer progression [59]. In an important insight, investigators focused also on the pathogenic mtDNA T8993G (within ATPase 6 of complex V) in prostate cancer and found that the T8993G mutation modifies mitochondrial ATPase synthesis. Importantly, these authors found that the mutant tumors also generated significantly more ROS, leading to an increase in DNA damage and hence tumor growth [9]. 

All these findings reveal that a few specific mitochondrial polymorphisms may be useful candidates for cancer biomarkers, but most somatic mitochondrial changes do not produce alterations in amino acids and their biologic functional contribution remains unclear. Mitochondria could play a causative role in an increasing risk of developing neoplastic lesions and progression, but further studies are required, perhaps by using experimental models including cybrids and analysis of large cohorts of patients. Furthermore, an accurate validation of tumor-associated mtDNA mutations by comparing normal and tumor samples followed by their detection in clinical samples should facilitate cancer prevention, early detection and therapeutic strategies. 

## MITOCHONDRIA AND BREAST CANCER

### D-loop Alterations

Several studies have focused on the association between mitochondrial variants and breast cancer risk. The majority of mutations were specifically detected in the D-loop, and in NADH dehydrogenase, cytochrome oxidase and ATPase genes [60-63]. In tumoral samples, the D-loop represents one of the most important mitochondrial "hotspot" regions, harboring a large number of alterations which are significantly associated to breast cancer (Table **1**). Among the alterations reported in literature over the past years, polycytidine stretch D310 (located between nucleotide 303 and 315 and interrupted by a T in position 310) has been frequently retrieved in tumoral samples, representing a potential starting point for the clonal expansion of malignant cells including breast cancer [64-71]. Indeed, D310 resulted the most significant mutation retrieved in breast samples [72].

Additional results have been reported about the role of the dinucleotide repeat polymorphism (CA)_n_ in carcinogenesis risk. Tseng *et al.* [73] indicated a very low prevalence of the CA deletion in 60 breast subjects (1.6%), whereas another study conducted on more than 1000 cases evidenced that D-loop (CA)_n_ polymorphism was not responsible for breast cancer risk but, conversely, should be associated with breast cancer survival [74].

**Fig. (1):**
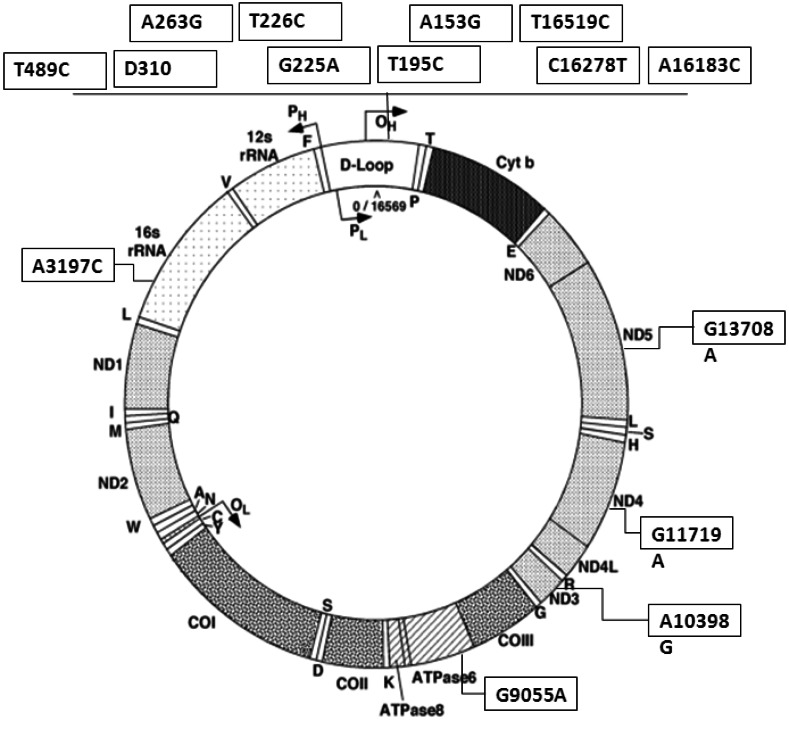
Map of variations in D-loop and PCGs associated with familial breast cancer [49, 75 and Tommasi personal communication].

Other studies have focused on a few important variants significantly retrieved in breast tumoral subjects, such as C16290T, 16293del-A and T16519C [75, 76]. In particular, the mutation T16519C in the D-loop was found to increase breast cancer risk (P= 0.0366), either occurring singularly or in association with other mitochondrial PCGs alterations such as A10398G, G13368A or C14766T [75, 77]. There are only few data which consider D-loop in familial breast cancer patients [49]; this region resulted more frequently altered than that in sporadic breast cancer and the variations which seem to be more present in familial breast cancer are A263G, T489C and D310. Furthermore, in our hands, seven further loci are specifically associated with breast cancer familiarity (Fig. **1**). Since scant data are available about the role of other mutations in breast carcinogenesis, further studies about the association between mitochondrial D-loop polymorphisms and breast cancer risk are needed.

### Mitochondrial PCGs Mutations

Recently, many authors have reported several alterations, detected in breast tissues, which are associated with cancer or mild breast pathologies [78, 79]. In particular, the most frequently mutated genes were *ND1*, *ND3*, *ND4* and *ND5* in a total of 6 missense substitutions and 5 synonymous alterations, of which 6 were significantly retrieved in breast cancer tissues (Table **1**). Specifically, in the last studies the mtDNA variant G10398A, which results in a non-conservative substitution of threonine for alanine within the *ND3* gene, has received the greatest attention. Many authors have reported that the presence of the 10398A allele has been significantly (P= 0.01) associated with an increased breast cancer risk in African-American women [80, 81]. Conversely, other studies found that mtDNA G10398A polymorphism did not result as a marker of breast cancer risk in African Americans, but individuals inheriting the A10398G polymorphism harbored a significant risk (P= 0.011) of developing breast cancer [77, 82, 83]. Additionally, an increased breast cancer risk (P= 0.03) associated with alcohol consumption was observed in a case-control study limited to carriers of the 10398G allele [84]. Moreover, the association of several variants resulted in a significant predictive breast cancer factor. Indeed, A10398G, together with some other mutations such as T4216C (P= 0.0009), G9055A (P= 0.0004), T16519C (P= 0.002) or A12308G (P= 0.0028), was found to increase the risk of a woman developing breast cancer [77, 85].

One of the most common somatic mtDNA deletions in tumoral cells (∆mtDNA^4977^) occurs between nucleotides 8.470 and 13.477 of the human mtDNA and includes 5 tRNA genes, 4 genes encoding subunits of NADH dehydrogenases, cytochrome oxidases subunit 3 (*CO3*) and ATPases genes. The major consequence is that ∆mtDNA^4977^ creates a smaller mtDNA molecule that leads to a decrease in energy production and to an abnormal ROS generation [86]. The ∆mtDNA^4977^, also called "the common deletion", seems to be associated with several mitochondrial encephalomyopathies including Pearson’s syndrome, Kearns-Sayre syndrome (KSS) and chronic progressive external ophthalmoplegia (CPEO) [87]. The "common deletion" has been studied in several human cells colonies [88-90] and also in liver [91] and breast tumors [73, 92].

The discovery that ∆mtDNA^4977^ occurs in low or discrete percentages in solid tumors, from 0.1% in colon tumors up to 30% in gastric and breast cancers [93] has led to controversial hypotheses. Some papers hypothesized that during carcinogenesis the cells containing mtDNA with no deletions are supported by a strong selection pressure compared to those with this severe alteration [91]. Other studies assumed that this deletion accumulates in many tissues during aging and has been used as a mtDNA damage biomarker [94]. Moreover, Shen and colleagues [95] found that ∆mtDNA^4977^ was implicated in the occurrence of breast and colorectal cancer, playing a role in modulating mtDNA contents in cancer cells.

Some researchers have performed a comparative analysis of expression levels of mitochondrial genes in benign and malignant breast tumors. Sharp *et al.* [96] found that the cytochrome *c *oxidase, subunit 2 (*CO2*) is expressed at significantly higher levels in carcinomas as compared to fibroadenomas, while no differences were found for ATPases or *ND2* and *ND4*. The only author [75] who considered mitochondrial PCGs alterations in familial compared to sporadic breast cancer patients showed a significant increased frequency of variations in 16S rRNA, ATP6, *ND3* and *ND5*; while the synonymous G11719A alteration in *ND4* gene has been significantly associated to familial breast cancer by our studies still unpublished (Fig. **1**).

Conclusively, even if many mitochondrial alterations have been detected and studied in breast cancer tissues, to date only a few mutations have been indicated as reliable biomarkers. One of these is the A10398G mutation which apparently remains one of the main independent predictors of the risk for breast carcinogenesis.

### Mitochondria and Breast Cancer Susceptibility Genes

Genetic linkage studies have identified *BRCA1* and *BRCA2* genes (chromosomes 17q21 and 13q12, respectively) as validated susceptibility markers of hereditary breast cancer (HBC). In particular, the *BRCA1* gene, when harboring germline mutations, confers a high susceptibility to breast and ovarian cancer predisposition and may account for a total of 10% of breast cancer incidence [97]. The Brca1 protein is involved in the control of genomic stability in the nucleus, such as in cell cycle regulation and checkpoint activation [98], by modulating specific transcriptional pathways and several highly specialized DNA repair processes [99, 100]. Brca1 is also implicated in the regulation of centrosomes, apoptosis, DNA binding and chromatin remodeling [101, 102]. 

Current advanced molecular technologies, including bi-directional sequencing and High Resolution DNA Melting Analysis, allow researchers to retrieve a widespread number of mutations in the *BRCA1/2* genes of patients with a familial history of breast cancer [103, 104]. All the pathogenic mutations and unclassified variants retrieved in *BRCA1/2* genes are reported in the Breast Cancer Core database (BIC: http://research.nhgri.nih.gov/bic). However, the pathogenic mutations found in *BRCA1/2* genes account for only ~40% of familial breast cancer cases and there is a wide cohort of subjects harboring wild-type *BRCA1/2* genes. It has therefore been suggested that other genes could be involved as predisposing factors to breast cancer [30, 105, 106]. Indeed, besides *BRCA *mutations, the current consensus is that modifications in *BRCA1/2* genes allow the accumulation of other cellular defects. In the most recent studies, *BRCA1/2* gene function in the DNA damage response pathway has led to the identification of a discrete number of susceptibility genes, including *ATM*, *BRIP1*, *CASP8, CHEK2*, *NBN*, *PALB2*, *PTEN*, *TP53* and *STK11* [29-31, 106-110].

Recently, genome wide association studies (GWAS) have also focused on the contribution of both *BRCA1/2* gene alterations and genetic modifiers which could increase hereditary predisposition to breast cancer [31]. In the general population, breast carcinogenesis may also be attributed to rare mutations of other genetic modifiers identified by GWAS (i.e., 2q35, 5p12 and 19p13) which confer a moderate risk of cancer development [31, 97].

Furthermore, many findings have highlighted an interesting relationship between the nucleus and mitochondria, as it has been demonstrated that the majority of mitochondrial proteins are nuclear encoded and post-translationally imported in mitochondria [111]. Coene and colleagues [112] evidenced the nuclear, cytoplasmic and mitochondrial localization of Brca1 proteins in human cells. In particular, mitochondrial Brca1 proteins seem to have an antiproliferative activity on breast cancer cells [113]. Finally, Bandiera *et al.* [114] found a large number of nuclear-encoded miRNAs, named as “mitomiRs”, which resulted differently expressed in mitochondria and cytosol, showing that most mitochondrial miRNAs were both nuclear and mitochondrial-encoded targets. The nuclear/mitochondrial connection way had also been previously demonstrated by a diagnostic algorithm showing that the mitochondrial deletion ∆mtDNA^4977^ in association to alterations in nuclear genes, such as *BRCA*, *ER *and *TP53 *genes, led to a phenotypic expression of premature aging and breast cancer [115].

Several other nuclear genes that impact upon the carcinogenic process have been identified specifically in mitochondria, such as sirtuin proteins (Sirt3) belonging to the deacetylases protein family. Since human breast and other human cancer specimens exhibit reduced Sirt3 levels, it has been suggested that sirtuins act as mitochondrial tumor suppressors, modulating both aging and tumoral phenotype [116, 117]. All these findings have been recently confirmed by an interesting study that showed that changes in mtDNA can produce different expression levels of specific nuclear-encoded genes (i.e. *MMP-9* and *Col1a*) which are capable of triggering the phenotype such as the one seen in malignant cells [118].

Moreover, the role of mitochondrial proteins in breast cancer development has been widely studied in recent years. A potential biomarker of the mitochondrial complex I subunit, *NDUFS*, resulted a strong indicator of breast cancer aggressiveness. It discriminated between normal and highly invasive breast carcinoma specimens, supporting a plausible mechanism involving mitochondrial dysfunction during the process of cancer cell transformation [119]. Moreover, a recent study found that the mitochondria of breast carcinoma expressed higher levels of mitochondrial fission protein dynamin-related protein 1 (Drp1), determining metastases to lymph nodes [37].

These findings show that nuclear mutated genes are responsible for only a part of hereditary breast cancer; the role played by mitochondrial modifiers in the general population highlighted the importance of these *loci* in breast carcinogenesis. Further studies should provide an opportunity to better understand the complicated relationship between genetic background and breast cancer etiology, describing the pathway through which they molecularly act.

### Mitochondrial Haplogroups and Breast Cancer

Some studies have focused on studying the risk resulting from the association between a mitochondrial haplogroup and breast carcinogenesis. In a study conducted on a total of 416 subjects, Bai *et al.* [75] suggested that individuals classified as haplogroup K show a significant increase in the risk of developing breast cancer (P= 0.0004), whereas individuals bearing haplogroup U have a significant decrease in breast cancer risk (P= 0.0023). 

The results obtained by Darvishi and colleagues [81] are more interesting. These authors analysed ~1000 complete human mtDNA sequences worldwide and 124 sporadic breast cancer patients from India, validating the exclusive presence of the pathogenic alteration G10398A (P= 0.01), which in literature is assigned to N haplotype. The apparent worldwide correlation in increased incidence rates of breast cancer and mtDNA haplogroup N distribution observed is interesting. These results were also confirmed by Gochhait *et al.* [120] who also observed the concomitant presence of 10398A in all 36 breast cancer patients in their study characterized by the N haplogroup.

Finally, Czarnecka *et al.* [61] concluded that haplogroup I assigned to 44 subjects among the Polish population is over-represented in individuals with breast cancer, whereas two studies agree that a total of 158 subjects from China harboring haplogroup M showed an increased carcinogenesis risk [121, 122].

In conclusion, mitochondrial dysfunction does appear to be a factor in cancer aetiology, an insight that may suggest new approaches for diagnosis and treatment. When comparing all the data on somatic mutations and haplogroup studies, no definitive results are provided by authors because the effect of the mitochondrial genetic background could be influenced by other features such as physiological conditions (i.e., hormonal state, age, sex) or geographical place of origin. Thus, the identification of significant mtDNA SNPs associated with breast cancer suggests that mitochondria may be involved in the pathogenetic mechanism of disease and cancer. To understand the etiology of the effect of mtDNA haplogroups or mitochondrial polymorphisms, many authors are looking for more nuclear and/or somatic mutations to determine if they play a critical role in breast carcinogenesis. Furthermore, mtDNA-SNPs association studies and haplogroup categorization are needed to obtain more pieces of this molecular puzzle.

## Figures and Tables

**Table 1.** Mitochondrial Variants Detected in Breast Cancer Tissues (nc= Noncoding; nr= Not reported; syn= Synonymous; *= Significant P-value).

Gene	Nucleotide Position	Nucleotide Change	Amino Acid Change	P-value	Reference
16S rRNA	3197	T>C	Nc	0.03*	Bai *et al.*, 2007
*ND1*	3918	G>A	Syn	nr	Parrella *et al.*, 2001
	4216	T>C	Y-->H	0.598	Covarrubias *et al.*, 2008
*ATP6*	9055	G>A	A-->T	0.005*	Bai *et al.*, 2007
*ND3*	10397	A>G	syn	0.030*	Fang *et al.*, 2010
	10398	A>G	T -->A	0.011*	Covarrubias *et al.*, 2008
	10400	C>T	syn	0.040*	Fang *et al.*, 2010
*ND4*	11719	G>A	syn	0.005*	Tommasi *et al.*, personal data
	11900	G>A	V-->M	nr	Parrella *et al.*, 2001
TL2	12308	A>G	nc	0.84	Covarrubias *et al.*, 2008
*ND5*	12344	T>A	M-->K	nr	Parrella *et al.*, 2001
	13708	G>A	A-->T	0.0006*	Bai *et al.*, 2007
*CYTB*	14869	G>A	syn	nr	Parrella *et al.*, 2001
*D-Loop*	16093	C>T	nc	nr	Parrella *et al.*, 2001
	16183	A>C	nc	0.03*	Tommasi *et al.*, personal data
	16278	C>T	nc	0.03*	Tommasi *et al.*, personal data
	16290	C>T	nc	0.002*	Sultana *et al.*, 2012
	16292	C>T	nc	0.663	Ma *et al.*, 2011
	16293	del-A	nc	0.010*	Sultana *et al.*, 2012
	16304	T>C	nc	0.252	Czarnecka *et al.*, 2010a
	16390	G>A	nc	1.00	Czarnecka *et al.*, 2010a
	16519	T>C	nc	0.036*	Bai *et al.*, 2007
	153	A>G	nc	0.009*	Tommasi *et al.*, personal data
	195	T>C	nc	0.04*	Tommasi *et al.*, personal data
	225	G>A	nc	0.03*	Tommasi *et al.*, personal data
	226	T>C	nc	0.03*	Tommasi *et al.*, personal data
	D310	insC	nc	<0.0001*	Xu *et al.*, 2012
